# Derivation and validation of plasma endostatin for predicting renal recovery from acute kidney injury: a prospective validation study

**DOI:** 10.1186/s13054-018-2232-5

**Published:** 2018-11-16

**Authors:** Hui-Miao Jia, Yue Zheng, Li-Feng Huang, Xin Xin, Wen-Liang Ma, Yi-Jia Jiang, Xi Zheng, Shu-Yan Guo, Wen-Xiong Li

**Affiliations:** Department of Surgical Intensive Care Unit, Beijing Chao-yang Hospital, 8 Gongren Tiyuchang Nanlu, Chaoyang District, Beijing, 100020 China

**Keywords:** Endostatin, Acute kidney injury, Renal recovery, Prognosis

## Abstract

**Background:**

Acute kidney injury (AKI) is associated with high morbidity and mortality in surgical patients. Nonrecovery from AKI may increase mortality and early risk stratification seems key to improving clinical outcomes. The aim of the current study was to explore and validate the value of endostatin for predicting failure to recover from AKI.

**Methods:**

We conducted a prospective cohort study of 198 patients without known chronic kidney disease who underwent noncardiac major surgery and developed new-onset AKI in the first 48 h after admission to the ICU. The biomarkers of plasma endostatin, neutrophil gelatinase-associated lipocalin (NGAL) and cystatin C were detected immediately after AKI diagnosis. The primary endpoint was nonrecovery from AKI (within 7 days). Cutoff values of the biomarkers for predicting nonrecovery were determined in a derivation cohort (105 AKI patients). Predictive accuracy was then analyzed in a validation cohort (93 AKI patients).

**Results:**

Seventy-six of 198 (38.4%) patients failed to recover from AKI onset, with 41 in the derivation cohort and 35 in the validation cohort. Compared with NGAL and cystatin C, endostatin showed a better prediction for nonrecovery, with an area under the receiver operating characteristic curve (AUC) of 0.776 (95% confidence interval (CI) 0.654–0.892, *p* < 0.001) and an optimal cutoff value of 63.7 ng/ml. The predictive ability for nonrecovery was greatly improved by the prediction model combining endostatin with clinical risk factors of Sequential Organ Failure Assessment (SOFA) score and AKI classification, with an AUC of 0.887 (95% CI 0.766–0.958, *p* < 0.001). The value of the endostatin–clinical risk prediction model was superior to the NGAL-clinical risk and cystatin C-clinical risk prediction models in predicting failure to recover from AKI, which was supported by net reclassification improvement and integrated discrimination improvement. Further, the endostatin–clinical risk prediction model achieved sensitivity and specificity of 94.6% (76.8–99.1) and 72.7% (57.2–85.0), respectively, when validated in the validation cohort.

**Conclusion:**

Plasma endostatin shows a useful value for predicting failure to recover from AKI. The predictive ability can be greatly improved when endostatin is combined with the SOFA score and AKI classification.

**Electronic supplementary material:**

The online version of this article (10.1186/s13054-018-2232-5) contains supplementary material, which is available to authorized users.

## Key messages

- AKI is associated with high morbidity and mortality in surgical patients. Nonrecovery from AKI increases mortality.
- Endostatin was able to help clinicians recognize the patients who failed to recover early at the time of diagnosing AKI. The predictive ability for nonrecovery from AKI can be greatly improved when endostatin is combined with the SOFA score and AKI classification.
- Starting individual treatments and effective interventions early in patients whose plasma endostatin is greater than 63.7 ng/ml or when the probability from the endostatin–clinical risk prediction model is greater than 0.279 at the time of diagnosing AKI may facilitate renal function recovery and reduce mortality.

## Background

Acute kidney injury (AKI) is a common postoperative complication and independently associated with high morbidity and mortality in surgical patients [1–3]. Nonrecovery from AKI has a negative impact on the prognosis of these patients, and greatly increases the risk for need of renal replacement therapy (RRT), persistent renal dysfunction, chronic kidney disease (CKD) and short-term and long-term mortality [4–6]. However, if the patients failing to recover can be identified early, individual treatments and effective interventions may be started at an early stage before real renal damage occurs and irreversible recovery happens, such as avoiding nephrotoxins, implementing volume management and individualized hemodynamic resuscitation [7, 8], which may decrease the classification of AKI, facilitate renal function recovery, reduce mortality and improve clinical outcomes. Plasma endostatin was recently discovered as a good biomarker for AKI prediction, which reflects renal structural damage in the early stage of AKI and elevates before creatinine increases [8, 9]. The predictive value of endostatin for AKI is considered to be superior to that of neutrophil gelatinase-associated lipocalin (NGAL) and cystatin C [9]. However, there has been no study exploring the value of endostatin for predicting nonrecovery from AKI [10, 11]. Surgical patients at high risk of AKI are prone to suffer from AKI soon after intensive care unit (ICU) admission. The current study aimed to evaluate and validate the utility of plasma endostatin for predicting nonrecovery in noncardiac postoperative AKI patients.

## Methods

The study was approved by the Human Ethics Committee of Beijing Chao-Yang Hospital, Capital Medical University (Beijing, China) (ethics number 2016-73). Written informed consent was obtained from patients or their next of kin before patients participated in this study.

### Study setting and population

The present study was performed in a 20-bed surgical ICU of Beijing Chao-yang Hospital from April 1, 2016 to July 31, 2017. The study design, performance and report complied with the Standards for Reporting of Diagnostic Accuracy guidelines [12]. We screened noncardiac postoperative patients who stayed in the ICU longer than 48 h. The patients diagnosed with AKI in the first 48 h after admission to the ICU were prospectively and consecutively enrolled. The exclusion criteria included: age < 18 years; developing AKI before ICU admission; acquired insufficient blood samples; and chronic kidney disease (CKD). All enrolled patients adhere to the following management principles: active treatment of primary disease and complications; and the same principles of treatment with antibiotics, nutritional metabolism and organ support.

### Biomarker measurements

Blood samples were collected immediately after AKI diagnosis. Acquired blood samples were rested for 30 min and subsequently centrifuged at 3000 rpm at 4 °C for 10 min, and supernatant plasma was stored and frozen at − 80 °C. Endostatin, NGAL and cystatin C in plasma were measured with a commercially available enzyme-linked immunosorbent assay (ELISA) kit (ab100508, Lot GR3183088-1 (endostatin); ab119600, Lot GR316206-1 (NGAL); ab179883, Lot GR308840–1 (cystatin C); Abcam, UK). The biomarkers were measured by technicians who were blind to clinical data and the physicians in charge were blind to the biomarker test results.

### Clinical endpoints and definitions

The primary endpoint was nonrecovery from AKI. Renal recovery was defined as not classifying for any creatinine-based AKI stage within 7 days (a serum creatinine level decreased to less than 150% of baseline from AKI onset) [13]. The patients using RRT until the 7th day after AKI were regarded as nonrecovery. Urine output-based criteria were not used because some patients were transferred to an ordinary ward 48 h after ICU admission and stayed in the ICU for less than 7 days. The urinary catheter might be sequentially removed. We cannot accurately measure hourly urine output for these patients in an ordinary ward. The secondary endpoints were ICU mortality, hospital mortality and 28-day mortality.

Major surgery is defined as a surgery classification of grade 3 or 4 identified by the National Health Commission of China. In this study, major surgery included esophagectomy, pulmonary lobectomy, gastrectomy, partial hepatectomy, pancreaticoduodenectomy, gastrointestinal perforation surgery, nephrolithotomy, cystectomy, orthopedic surgery and so forth. We can obtain the surgery classification directly from the electronic medical record system.

The diagnosis of AKI depended on the serum creatinine criteria proposed by Kidney Disease: Improving Global Outcomes (KDIGO) as either of the following: increase in serum creatinine by ≧ 3 mg/dl (≧ 26.5 μmol/l) within 48 h; or increase in serum creatinine to ≧ 1.5 times baseline, which is known or presumed to have occurred within the prior 7 days [14, 15]. Baseline creatinine was defined as follows: if at least five values were available, the median of all values available from 6 months to 6 days prior to enrollment was used. Otherwise, the lowest value in the 5 days prior to enrollment was used [16]. If no preenrollment creatinine was available or the emergency patient’ s serum creatinine was abnormal at the time of admission, the baseline creatinine was estimated using the Modification of Diet in Renal Disease (MDRD) equation assuming that the baseline estimated glomerular filtration rate (eGFR) is 75 ml/min per 1.73 m^2^ [17]. CKD was defined according to the definition of the National Kidney Foundation as eGFR < 60 ml/min/1.73 m^2^ for at least 3 months irrespective of the cause. The GFR was estimated with the Cockcroft–Gault formula [17, 18].

### Sample size calculation

The formula calculating the sample size for a cohort study was used in this study:

$$ \mathrm{n}=\frac{{\left({Z}_{\alpha}\sqrt{2\overline{\mathrm{p}\mathrm{q}}}+{Z}_{\beta}\sqrt{{\mathrm{p}}_0{\mathrm{q}}_0+{\mathrm{p}}_1{\mathrm{q}}_1}\right)}^2}{{\left({\mathrm{p}}_1\hbox{-} {\mathrm{p}}_0\right)}^2}, $$

where Z is a statistical value, p_1_ and p_0_ represent the expected incidence of the exposure group and the nonexposure group, respectively, q_0_ = 1– p_0_, q_1_ = 1 – p_1_, $\overline{\mathrm{p}}$ is the average of the two incidence, $\overline{\mathrm{q}}$ = 1 – $\overline{\mathrm{p}}$, α = 0.05 and the power (1 – β) is 90%.

According to our pretest results the incidence of nonrenal function recovery was 0.49 in the exposed group (plasma endostatin level above the threshold) and 0.16 in the nonexposed group (plasma endostatin level below the threshold). According to the presented formula, the sample size of the derivation cohort calculated was 82. The same formula was used to calculate the validation cohort sample size, which was also 82. Therefore, the sample size of this study is derivation cohort + validation cohort = 164. Considering the lost rate to follow-up (about 10%), the estimated total sample size was 164 + (164 × 10%) = 181.

### Data collection

All clinical data were prospectively collected on the basis of case report forms (CRF). Serum creatinine was detected and recorded at ICU admission and every 12 h thereafter until the 7th day after AKI. Severity of patient illness was estimated by the Acute Physiology and Chronic Health Evaluation (APACHE II) and Sequential Organ Failure Assessment (SOFA) scores on the day of diagnosing AKI. Clinical variables containing patient demographic characteristics, prior health history, diagnosis, surgery procedure, duration of mechanical ventilation, ICU stay and hospital stay were collected from the electronic medical record system.

### Study phase

The study contained two phases. Phase I (derivation cohort) was performed from April 1, 2016 to December 31, 2016. Data from these patients were used to determine the cutoff value of plasma endostatin which best discriminated AKI patients with or without renal recovery. Phase II (validation cohort) was performed from January 1, 2017 to July 31, 2017. Using the cutoff values previously determined in the derivation cohort, the predictive accuracy of plasma endostatin in predicting nonrecovery was thus evaluated in the validation cohort.

### Statistical analysis

SPSS statistics 24 (IBM, Chicago, IL, USA) and R 2.1.2 were used for statistical analyses. Continuous variables were presented as mean ± standard deviation (SD) or median (25th, 75th percentiles), categorical variables were presented as percentiles. Continuous data between two groups (recovery group and nonrecovery group) were compared using the repeated measurement analysis of variance or Mann–Whitney *U* tests, and categorical variables used the chi-square test or Fisher’ s exact test. For all analyses, statistical significance was indicated by two-sided *p* < 0.05.

In the derivation cohort, the predictive values of the biomarkers for nonrecovery from AKI were assessed by the receiver operating characteristic (ROC) curve. The area under the ROC curve (AUC), sensitivity, specificity and their corresponding 95% confidence intervals (CIs) as well as cutoff biomarker values for predicting nonrecovery were recorded. The following values were used to describe AUCs: 0.90–1.0, excellent; 0.80–0.89, good; 0.70–0.79, useful; 0.60–0.69, poor; and 0.50–0.59, no useful performance [9]. The optimal cutoff values were estimated as those that minimized false negatives (i.e., patients who were not identified as nonrecovery by the novel biomarkers but were identified as nonrecovery by the creatinine criteria), with specificity not lower than 50%. Furthermore, a combination of the ROC curve with multivariate logistic regression analysis was used to assess the predictive value of biomarkers and clinical parameters for nonrecovery. Clinical parameters with *p* < 0.1 in univariate analyses were added to the multivariate logistic regression model. The net contribution of the biomarkers to predict renal recovery was validated by Hosmer and Lemeshow’s test, net reclassification improvement (NRI) and integrated discrimination improvement (IDI).

In the validation cohort, predictive accuracy of the biomarker was assessed by sensitivity, specificity, positive predictive value (PPV) and negative predictive value (NPV), which were calculated by the true incidence of nonrecovery in the validation cohort.

## Results

### Total patient characteristics

**Fig. 1:**
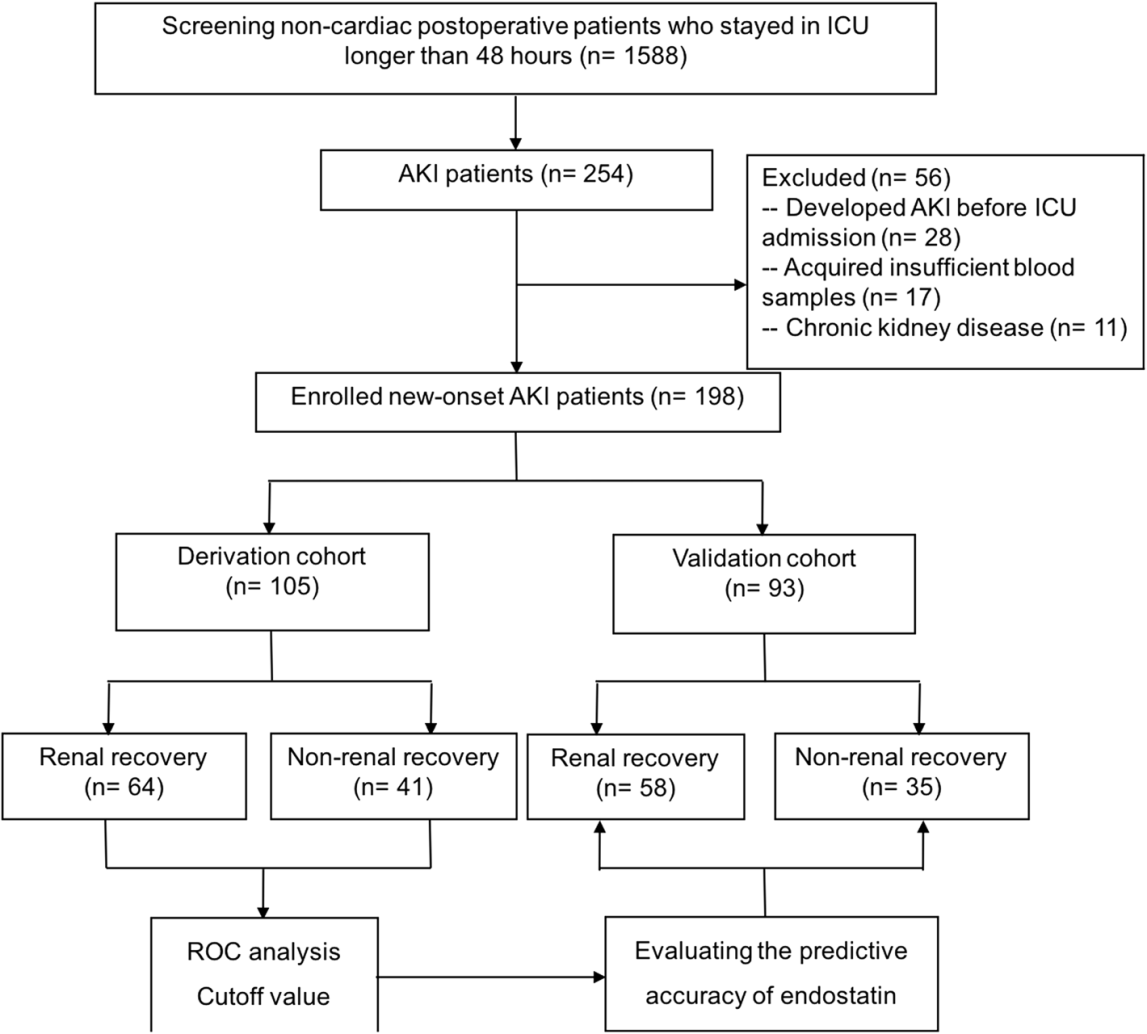
Study flow diagram. *AKI* acute kidney injury, *ICU* intensive care unit, *ROC* receiver operating characteristic

During the study period, 1588 patients who underwent noncardiac major surgery were screened, among them 254 (16.0%) patients developed AKI in the first 48 h after admission to ICU. After excluding the ineligible patients, 198 were finally enrolled, with 105 in the derivation cohort and 93 in the validation cohort. Baseline characteristics, severity of kidney injury and short-term mortality between the two cohorts are comparable with no significant difference. The data are presented in Table 1. The screening diagram is shown in Fig. 1.

**Table 1:** Patient baseline characteristics in derivation and validation cohorts

Variable	Derivation cohort (*n* = 105)	Validation cohort (*n* = 93)	*p* value
Baseline characteristics			
Age (years)	65 (52, 73)	64 (50, 67)	0.763
Female gender	45 (42.8)	36 (38.7)	0.518
BMI (kg/m^2^)	22.7 (19.9, 24.5)	23.9 (20.5, 26.1)	0.812
APACHE II score	14.9 (13.8, 17.0)	15.7 (14.0, 19.0)	0.634
SOFA score	6 (4, 7)	6 (5, 7.8)	0.663
Serum creatinine before surgery (μmol/L)	63.2 (54.0, 69.8)	66.5 (55.1, 73.4)	0.719
Hemoglobin (g/L)	86.0 (77.5, 97.0)	87.8 (78.4, 99.2)	0.826
Surgery			
Abdominal surgery	72 (69.6)	58 (62.3)	0.497
Contaminated surgery procedure	70 (57.1)	52 (55.9)	0.672
Duration (h)	5.2 (2.2, 8.7)	5.4 (2.5, 7.9)	0.582
Bleeding (ml)	450 (200, 800)	500 (250, 900)	0.439
Major infection site			
Intra-abdominal infection	18 (17.1)	14 (15.1)	0.619
Pulmonary infection	5 (4.7)	8 (8.6)	0.524
Bloodstream infection	9 (8.6)	5 (5.4)	0.587
Urinary tract infection	8 (7.6)	9 (5.4)	0.496
Others	6 (5.7)	7 (7.5)	0.703
Serum creatinine diagnosing AKI (μmol/L)	137.4 (118.3, 158.4)	142.6 (112.5, 164.7)	0.413
UO 24 h after ICU admission (ml/kg/h)	1.0 (0.5, 1.5)	1.1 (0.7, 1.8)	0.576
UO 48 h after ICU admission (ml/kg/h)	0.8 (0.4, 1.9)	0.9 (0.4, 1.5)	0.618
AKI classification			
Stage 1	42 (40.0)	36 (38.7)	0.672
Stage 2	44 (41.9)	41 (44.0)	0.754
Stage 3	19 (18.1)	16 (17.3)	0.812
Outcome			
Renal recovery in 7 days	64 (60.9)	58 (62.3)	0.645
Renal recovery at hospital discharge	69 (65.7)	62 (66.6)	0.783
Need for RRT in 7 days	19 (18.1)	18 (19.3)	0.796
Hospital mortality	9 (9.2)	8 (8.6)	0.510
28-day mortality	14 (13.3)	12 (12.9)	0.694

Values are median (25th, 75th percentile interquartile range) or *n* (%)

*AKI* acute kidney injury, *APACHE II* acute physiology and chronic health evaluation, *BMI* body mass index, *ICU* intensive care unit, *RRT* renal replacement therapy, *SOFA* sequential organ failure assessment, *UO 24 h* first 24-h urine output, *UO 48 h* first 48-h urine output

### Derivation cohort characteristics

In the derivation cohort, 59 (56.2%) had renal recovery and 46 (43.8%) patients failed to recover from AKI onset. Patients with and without renal recovery showed no significant difference in the baseline serum creatinine, chronic comorbidities, infection status, first 24-h and 48-h urine output, surgery data and fluid balance in surgery. However, patients who failed to recover had more serious illness with higher APACHE II and SOFA scores on the day of diagnosing AKI. Further, AKI stage 1 and 3 showed great statistical difference between patients with and without recovery. Significant difference of plasma endostatin, NGAL and cystatin C was also observed. Recovery patients showed concentrations of 62.6 (48.3, 87.6) ng/ml, 119.0 (105.4, 200.2) ng/ml and 4.8 (2.7, 9.2) mg/dl, respectively, whereas patients failing to recover showed higher concentrations of 108.5 (71.4, 163.8) ng/ml, 149.8 (110.1, 256.2) ng/ml and 8.7 (5.6, 13.2) mg/dl, respectively. These characteristic comparisons of patients with and without renal recovery are summarized in Table 2.

**Table 2:** Baseline characteristics between AKI patients with and without renal recovery in the derivation cohort

Variable	Recovery (*n* = 64)	Nonrecovery (*n* = 41)	*p* value
Baseline characteristics			
Age (years)	66 (50, 75)	61 (52, 68)	0.323
Female gender	32 (49.6)	13 (33.3)	0.349
BMI (kg/m^2^)	23.3 (20.9, 25.8)	24.3 (21.7, 27.4)	0.436
APACHE II score	14.0 (12.0, 16.0)	16.0 (14.0, 18.0)	0.018
SOFA score	5 (3, 6)	7 (5.8, 8)	0.001
Serum creatinine before surgery (μmol/L)	64.5 (56.4, 71.6)	65.1 (51.2, 72.6)	0.884
Hemoglobin (g/L)	85.0 (73.0, 91.5)	89.0 (83.0, 100.0)	0.199
Comorbidities			
COPD/asthma	8 (11.9)	4 (9.9)	0.709
Cardiovascular disease	12 (19.7)	13 (30.9)	0.319
Chronic liver disease	20 (31.6)	20 (49.4)	0.197
Cancer	21 (32.5)	20 (49.4)	0.155
Diabetes	22 (34.2)	16 (38.3)	0.679
Hypertension	30 (47.9)	20 (48.1)	0.720
Major infection site			
Intra-abdominal infection	9 (14.1)	9 (21.9)	0.354
Pulmonary infection	3 (4.6)	2 (4.9)	0.716
Bloodstream infection	4 (6.3)	5 (12.2)	0.375
Urinary tract infection	3 (4.6)	5 (12.2)	0.302
Others	3 (4.6)	3 (7.3)	0.583
Surgery			
Abdominal surgery	49 (76.1)	28 (69.1)	0.491
Contaminated surgery procedure	39 (60.9)	21 (51.2)	0.326
Duration (h)	5.0 (2.4, 8.7)	5.7 (2.7, 8.6)	0.561
Bleeding (ml)	300 (150, 900)	750 (50, 1450)	0.245
Fluid balance in surgery (ml)	2792 (1350, 4570)	2800 (1920, 4550)	0.899
Blood product transfusion			
Red blood cells (ml)	0 (0, 800)	400 (0, 2100)	0.329
Plasma (ml)	0 (0, 800)	400 (0, 900)	0.233
Serum creatinine diagnosing AKI (μmol/L)	119.7 (100.7, 133.5)	168.3 (126.9, 212.8)	< 0.001
UO 24 h after ICU admission (ml/kg/h)	1.1 (0.5, 1.8)	0.8 (0.3, 1.5)	0.243
UO 48 h after ICU admission (ml/kg/h)	1.0 (0.3, 1.8)	0.7 (0.2, 1.6)	0.289
AKI classification			
Stage 1	35 (54.6)	8 (19.5)	0.001
Stage2	23 (35.9)	20 (48.8)	0.458
Stage3	6 (9.3)	13 (31.7)	0.007
Endostatin (ng/ml)	62.6 (48.3, 87.6)	108.5 (71.4, 163.8)	< 0.001
NGAL (ng/ml)	119.0 (105.4, 200.2)	149.8 (110.1, 256.2)	0.045
Cystatin C (mg/dl)	4.8 (2.7, 9.2)	8.7 (5.6, 13.2)	0.029

Values are median (25th, 75th percentile interquartile range) or *n* (%)

*AKI* acute kidney injury, *APACHE II* acute physiology and chronic health evaluation, *BMI* body mass index, *COPD* chronic obstructive pulmonary disease, *ICU* intensive care unit, *NGAL* neutrophil gelatinase-associated lipocalin, *SOFA* sequential organ failure assessment, *UO 24 h* the first 24 h urine output, *UO 48 h* the first 48 h urine output

### Outcomes

ICU stay showed great difference between patients with and without renal recovery from AKI. Nonrecovery patients had longer ICU stay (10 (6.7–16.5) days vs 6 (4.0–10.0) days, *p* = 0.028) than recovery patients. ICU mortality, hospital mortality and 28-day mortality tended to be higher in nonrecovery patients than in recovery patients: mortalities were 3 (4.7%) vs 6 (14.6%) (*p* = 0.080), 3 (4.7%) vs 7 (17.1%) (*p* = 0.045) and 4 (6.3%) vs 10 (24.3%) (*p* = 0.008), respectively, despite the ICU mortality not reaching great statistical significance. The outcome data are presented in Table 3.

**Table 3:** Outcomes between AKI patients with and without renal recovery in the derivation cohort

Variable	Recovery (*n* = 64)	Nonrecovery (*n* = 41)	*p* value
MV (h)	35 (11.2, 60)	80.5 (24, 103)	0.061
ICU stay (days)	6 (4.0, 10.0)	10 (6.7, 16.5)	0.028
Hospital stay (days)	19 (13.5, 28.5)	24 (13.5, 37)	0.144
ICU mortality	3 (4.7)	6 (14.6)	0.080
Hospital mortality	3 (4.7)	7 (17.1)	0.045
28-day mortality	4 (6.3)	10 (24.3)	0.008

Values are median (25th, 75th percentile interquartile range) or *n* (%)

*AKI* acute kidney injury, *ICU* intensive care unit, *MV* mechanical ventilation

### Predicting nonrecovery from AKI in the derivation cohort

On univariate analysis, the APACHE II score, SOFA score, serum creatinine diagnosing AKI and AKI stage 1 and 3 showed an association with nonrecovery and were included in a clinical risk prediction model. This model predicted nonrecovery from AKI with an AUC of 0.782 (95% CI 0.661–0.895, *p* < 0.001). Because there was a positive linear correlation between APACHE II and SOFA scores (*r* = 0.496, *p* < 0.001), and serum creatinine diagnosing AKI and AKI classification (*r* = 0.547, *p* < 0.001), respectively, the SOFA score and AKI classification with better predictive value instead of the APACHE II score and serum creatinine diagnosing AKI were added to the model.

**Fig. 2:**
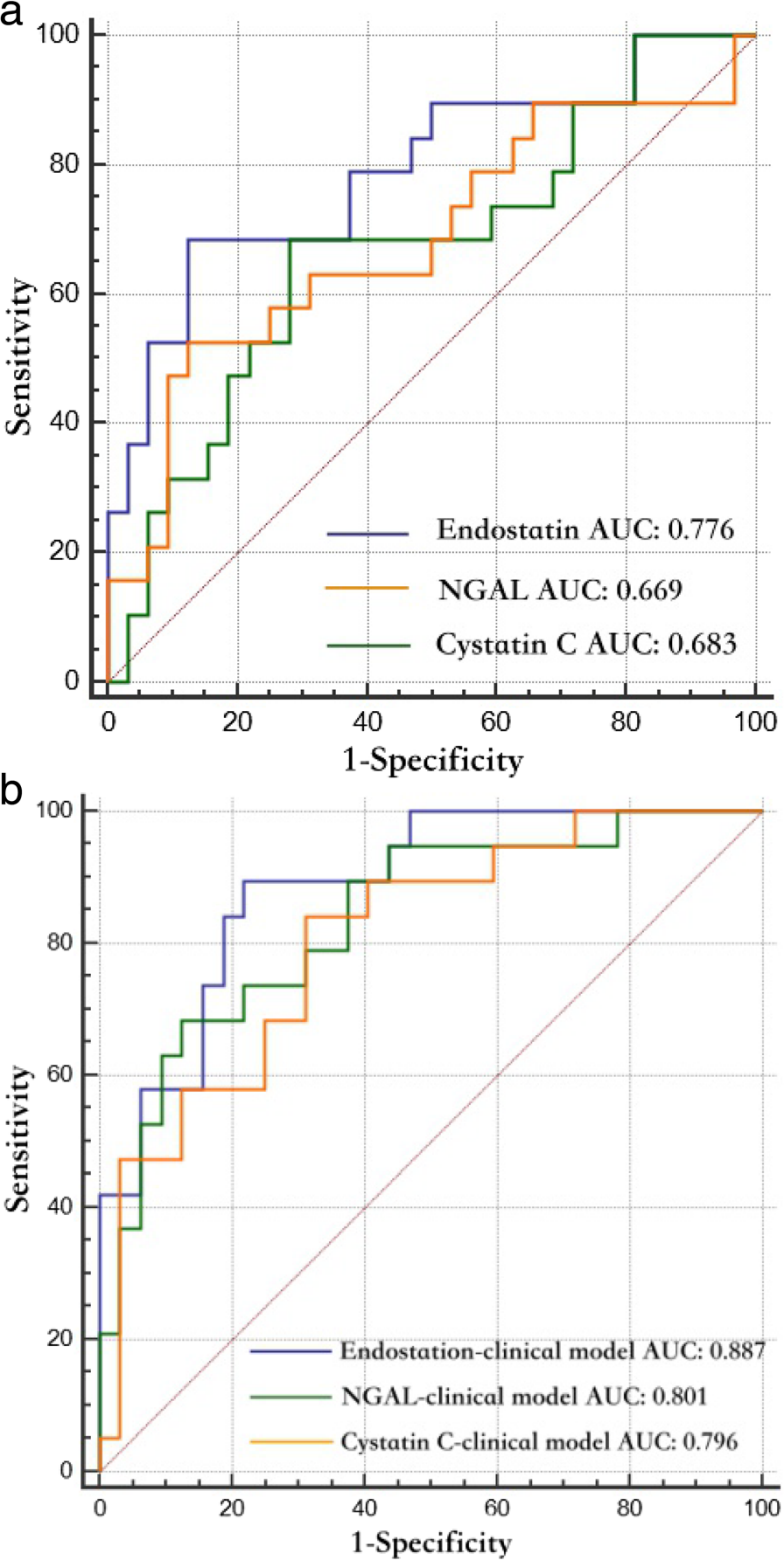
Predictive value of biomarkers and their corresponding models. ROC curves of endostatin, NGAL, cystatin C and their corresponding models for predicting failure to recover in derivation cohort. **a** AUCs of endostatin, NGAL and cystatin C alone for prediction. **b** AUCs of biomarker–clinical risk prediction models for prediction. *ROC* receiver operating characteristic, *AUC* area under the ROC, *NGAL* neutrophil gelatinase-associated lipocalin

Plasma endostatin alone yielded AUC of 0.776 (95% CI 0.654–0.892, *p* < 0.001) for predicting nonrecovery from AKI with an optimal cutoff value of 63.7 ng/ml. The predictive power for nonrecovery was significantly improved by combing endostatin with a clinical risk prediction model. The predictive AUC increased to 0.887 (95% CI 0.766–0.958, *p* < 0.001), confirmed by Hosmer and Lemeshow’s test (*p* > 0.05). In contrast, neither NGAL nor cystatin C alone showed useful predictive value for nonrecovery, with AUC of 0.669 (95% CI 0.524–0.795, *p* = 0.046) and 0.683 (95% CI 0.537–0.806, *p* = 0.037), respectively. Furthermore, the value of the endostatin–clinical risk prediction model was superior to those of the NGAL–clinical risk and cystatin C–clinical risk prediction models in predicting nonrecovery from AKI, which was supported by both NRI (endostatin vs NGAL *p* = 0.027, endostatin vs cystatin C *p* = 0.044) and IDI (endostatin vs NGAL *p* = 0.021, endostatin vs cystatin C *p* = 0.006) analysis (Additional file 1: Table S1). Multivariate logistic regression analysis calculated the probability for nonrecovery based on the endostatin–clinical risk prediction model: the probability for nonrecovery = 1 / (1 + e^–*z*^), *z* = − 4.029 + 0.022 × endostatin + 0.280 × SOFA score – 0.937 × AKI stage 1 + 1.850 × AKI stage 3. The optimal cutoff probability value was 0.279. AKI patients who have a probability value greater than 0.279 may fail to recover. The predictive performance of the biomarkers and combination models is presented in Table 4, and their ROC curves are presented in Fig. 2.

**Table 4:** Biomarkers and combination models for predicting nonrecovery from AKI

	AUC (95% CI)	Cutoff value	*p* value
Endostatin (ng/ml)	0.776 (0.654–0.892)	63.7	< 0.001
NGAL (ng/ml)	0.669 (0.524–0.795)	162.2	0.046
Cystatin C (mg/dl)	0.683 (0.537–0.806)	4.87	0.037
Clinical risk prediction model	0.782 (0.661–0.895)	0.259	< 0.001
Endostatin–clinical risk prediction model	0.887 (0.766–0.958)	0.279	< 0.001
NGAL–clinical risk prediction model	0.801 (0.707–0.926)	0.266	< 0.001
Cystatin C–clinical risk prediction model	0.796 (0.678–0.906)	0.286	< 0.001

*AKI* acute kidney injury, *AUC* area under the receiver operating characteristic, *CI* confidence interval, *NGAL* neutrophil gelatinase-associated lipocalin

### Predictive accuracy of endostatin for predicting nonrecovery from AKI in the validation cohort

In the validation cohort, the incidence of nonrecovery was 35/93 (37.6%). Using cutoff values selected in the derivation cohort, predictive accuracy was evaluated in the validation cohort. Compared with NGAL and cystatin C alone, endostatin alone showed the best predictive value for nonrecovery with sensitivity, specificity, PPV, NPV and their 95% CIs of 88.6% (69.8–97.6), 53.7% (40.7–67.5), 52.3% (39.2–66.8) and 88.5% (39.2–66.8), respectively. When endostatin was combined with clinical risk factors building the prediction model of *p* = 1 / (1 + e^–*z*^), *z* = – 4.029 + 0.022 × endostatin + 0.280 × SOFA score – 0.937 × AKI stage 1 + 1.850 × AKI stage 3 for predicting failure to recover, the sensitivity greatly improved to 94.6% (76.8–99.1) and the specificity, PPV and NPV were 72.7% (57.2–85.0), 66.7% (50.3–81.4) and 96.2% (82.3–100.0), respectively. The assessment of predictive accuracy for nonrecovery from AKI is presented in Table 5.

**Table 5:** Predictive accuracy of the biomarkers for nonrecovery

	Cutoff value	Sensitivity	Specificity	PPV	NPV
Endostatin (ng/ml)	63.7	88.6 (69.8–97.6)	53.7 (40.7–67.5)	52.3 (39.2–66.8)	88.5 (39.2–66.8)
NGAL (ng/ml)	116.2	61.5 (40.6–79.8)	52.7 (38.7–67.5)	41.7 (35.2–59.2)	67.6 (50.1–82.6)
Cystatin C (mg/dl)	4.87	65.4 (44.3–82.8)	50.0 (38.6–64.5)	43.6 (30.8–60.4)	71.0 (52.0–85.8)
Endostatin–clinical risk prediction model	0.279	94.6 (76.8–99.1)	72.7 (57.2–85.0)	66.7 (50.3–81.4)	96.2 (82.3–100.0)
NGAL–clinical risk prediction model	0.266	89.5 (66.9–98.7)	62.5 (43.7–78.9)	58.6 (40.9–76.5)	90.9 (70.8–98.9)
Cystatin C–clinical risk prediction model	0.286	88.4 (66.9–98.7)	57.6 (40.2–74.5)	54.8 (38.8–72.7)	90.2 (69.6–98.3)

Data presented as percentage (95% confidence interval)

*NGAL* neutrophil gelatinase-associated lipocalin, *NPV* negative predictive value, *PPV* positive predictive value

### Sensitivity analysis

Since serum creatinine is the diagnostic criterion for AKI classification and also a component of the SOFA score, the risk prediction analyses were repeated after removing AKI stage 1 and 3 from the clinical model. The predictive value of the model combining SOFA score and endostatin was identified to be steady and good, which was confirmed by Hosmer and Lemeshow’s test (*p* > 0.05), (Additional file 1: Table S2).

## Discussion

Persistent AKI may develop CKD or end-stage kidney disease (ESKD) with dialysis dependence, which strongly increased the risk for short-term and long-term mortality [19–22]. The current focus has turned toward the promotion of renal function recovery during the phase of kidney impairment [23, 24]. Diverse platforms are expected to find a novel biomarker to predict renal recovery from AKI. This is the first study to evaluate the ability of plasma endostatin for predicting failure to recover. The main findings were: endostatin alone had a useful value for predicting failure to recover from AKI, and showed a better predictive ability than NGAL and cystatin C; endostatin was validated to be able to help clinicians recognize the patients who failed to recover early at the time of diagnosing AKI, with sensitivity and specificity of 88.6% and 53.7%, respectively; the logistic regression model including endostatin and clinical risk factors greatly improved the predictive ability for nonrecovery, with a maximum AUC of 0.887—the utility of the model was validated in the diverse noncardiac postoperative AKI population, with sensitivity and specificity of 94.6% and 72.7%, respectively; and the patients who failed to recover had a worse short-term prognosis than the recovery patients.

In a multicenter, prospective, cohort study, plasma NGAL showed its ability for predicting failure to recover in patients with pneumonia-induced severe acute kidney injury [25]. The authors used the RIFLE criteria to diagnose AKI and tested plasma NGAL on the first day of RIFLE-F. The results indicated that plasma NGAL appeared to be a useful biomarker for predicting nonrecovery. In our study, endostatin performed with a better prediction value than NGAL so it seems that endostatin is a more promising biomarker to predict failure to recover, despite the different population, diagnostic criteria of AKI and predictive time window.

Endostatin is the C-terminal fragment of collagen XVII, produced by cleavage of collagen XVII during extracellular matrix remodeling [26]. The release of endostatin is triggered by several proteases [27]. After the proteolytic process, the endostatin in production can be released into circulation. Collagen XVII is a major constituent in the basement membranes and highly expressed in the renal tubular epithelium, Bowman’s capsule and glomerular basal membrane [28]. Recent studies observed the altered expression of endostatin preceding the kidney damage, and found the involvement of endostatin in the physiological response of renal impairment [28, 29]. Further, endostatin was thought to be associated with cytokine-mediated inflammation factors of C-reactive protein and IL-6 [30], which means endostatin has potential to participate in the pathophysiological inflammatory processes in renal damage, the development and progression of AKI. In a mouse model of ischemia/reperfusion (I/R)-induced AKI, endostatin mRNA and protein were upregulated and involved in the endothelial response to renal injury [31, 32]. Ischemia for 45 min may cause the process of proteolytic cleavage of collagen XVII and strong release of endostatin [33]. Elevated plasma concentration of endostatin is closely associated with deteriorated renal function. Endostatin rising early may indicate the progression of kidney injury.

In a previous study by Mårtensson et al. [9], plasma endostatin was tested for predicting AKI in critically ill patients. The results showed that endostatin was a good predictive factor and improved AKI prediction based on clinical risk factors. In a study by Ruge et al. [34], circulating endostatin was considered to parallel kidney damage and be involved in the development of CKD. They detected circulating endostatin in two community-based cohorts of elderly individuals and proved the hypothesis that the circulating marker levels were associated with damaged eGFR and predictive factors for the development of CKD. Further, a study evaluated the association between high endostatin level and mortality in patients with ESRD. However, a low relative risk was found between endostatin and long-term mortality [35]. Being different from the previous studies, the present study focused on noncardiac postoperative patients with new-onset AKI and tested plasma endostatin for predicting nonrecovery from AKI. Endostatin performed well in predicting the patients who failed to recover, especially when combined with clinical risk factors. Furthermore, if the plasma endostatin of the patient is greater than 63.7 ng/ml or the probability from the endostatin–clinical risk prediction model is greater than 0.279 at the time of diagnosing AKI, clinicians should consider starting individual treatments and effective interventions early. In this way, renal function may be turned to recovery and mortality may be reduced. Further clinical trials are still needed to evaluate the value of endostatin in predicting CKD development and the association between endostatin and prognosis of AKI patients.

Our study does have important limitations. The included patients were all noncardiac postoperative and cared for in a single center that may be different from other institutions. The predictive value of endostatin for renal recovery needs to be further assessed in different AKI populations with larger sample sizes. CKD is a risk factor for AKI, so it is important to evaluate AKI patients with worsening of preexisting CKD. This study only focused on new-onset AKI patients without known CKD. AKI patients with worsening of preexisting CKD also need further studies to explore the predictive value of endostatin for nonrenal recovery. Furthermore, it would be helpful for clinics to explore the association between endostatin with long-term prognosis of AKI. However, we did not provide an investigation of the long-term prognosis of these AKI patients because many patients were lost to follow-up.

## Conclusion

Plasma endostatin shows a useful value for predicting failure to recover from AKI. The predictive ability can be greatly improved when endostatin is combined with the SOFA score and AKI classification.

## Additional file

Additional file 1: **Table S1.** Net reclassification improvement (NRI) and integrated discrimination improvement (IDI) for assessing contributions of different biomarkers for nonrecovery prediction when combined with clinical risk factors. **Table S2.** Sensitivity analysis. Risk prediction analyses repeated after removing AKI stage 1 and 3 from clinical model. (PDF 41 kb)

## Abbreviations

AKI: Acute kidney injury
APACHE II: Acute Physiology and Chronic Health Evaluation
AUC: Area under the receiver operating characteristic curve
CI: Confidence interval
CKD: Chronic kidney disease
GFR: Glomerular filtration rate
ICU: Intensive care unit
IDI: Integrated discrimination improvement
KDIGO: Kidney Disease Improving Global Outcomes
NGAL: Neutrophil gelatinase-associated lipocalin
NPV: Negative predictive value
NRI: Net reclassification improvement
PPV: Positive predictive value
RIFLE: Risk, Injury, Failure, Loss, End-stage
RRT: Renal replacement therapy
SD: Standard deviation
SOFA: Sequential Organ Failure Assessment
