# Supplementary material for: Derivation and validation of plasma endostatin for predicting renal recovery from acute kidney injury: a prospective validation study
Source: Crit Care. 2018 Nov 16;22:305. doi: 10.1186/s13054-018-2232-5 (PMC6240328; doi:10.1186/s13054-018-2232-5)
Supplement: Supplementary file 1 — Table S1. Net reclassification improvement (NRI) and integrated discrimination improvement (IDI) for assessing contributions of different biomarkers for nonrecovery prediction when combined with clinical risk factors. Table S2. Sensitivity analysis. Risk prediction analyses repeated after removing AKI stage 1 and 3 from clinical model. (PDF 41 kb) [file 13054_2018_2232_MOESM1_ESM.pdf]

Supplementary Material

Table S1 NRI and IDI for assessing the contributions of different biomarkers for non-recovery prediction when combining with clinical risk factors

Biomarker model	NRI	*P* value	IDI	*P* value
Endostatin-clinical model vs. NGAL-clinical model	0.252	0.027	0.089	0.021
Endostatin-clinical model vs. Cystatin C- clinical model	0.228	0.034	0.102	0.006

The predictive value of endostatin-clinical risk model was superior to NGAL- and cystatin C-clinical risk model in predicting non-recovery from AKI. *AKI* acute kidney injury, *NRI* net reclassification improvement, *IDI* integrated discrimination improvement, *NGAL* neutrophil gelatinase-associated lipocalin.

Table S2 Sensitivity analysis

	AUC (95 % CI)	*p* value
SOFA + AKI stage 1 and 3	0.782 (0.661, 0.895)	< 0.001
SOFA	0.703 (0.589, 0.812)	< 0.001
Endostatin + SOFA + AKI stage 1 and 3	0.887 (0.766, 0.958)	< 0.001
Endostatin + SOFA	0.829 (0.701, 0.870)	< 0.001
NGAL + SOFA + AKI stage 1 and 3	0.801 (0.707, 0.926)	< 0.001
NGAL + SOFA	0.750 (0.638, 0.857)	< 0.001
Cystatin C + SOFA + AKI stage 1 and 3	0.796 (0.678, 0.906)	< 0.001
Cystatin C + SOFA	0.748 (0.629, 0.846)	< 0.001

The risk prediction analyses were repeated after removing AKI stage 1 and 3 from the clinical model. The predictive value of the model combining SOFA score and endostation was identified to be steady and good whether it is with and without AKI stage 1 and 3. *AUC* area under the receiver operating characteristic, *CI* confidence interval, *AKI* acute kidney injury, *SOFA* sequential organ failure assessment, *NGAL* neutrophil gelatinase-associated lipocalin.
